# Elevated MR-proANP plasma concentrations are associated with sepsis and predict mortality in critically ill patients

**DOI:** 10.1186/s12967-019-02165-2

**Published:** 2019-12-12

**Authors:** Eray Yagmur, Johanna Hermine Sckaer, Ger H. Koek, Ralf Weiskirchen, Christian Trautwein, Alexander Koch, Frank Tacke

**Affiliations:** 1 Medical Care Center, Dr. Stein and Colleagues, Tomphecke 45, 41169 Mönchengladbach, Germany; 2 Department of Medicine III, RWTH-University Hospital Aachen, Aachen, Germany; 3 Section of Gastroenterology and Hepatology, Department of Internal Medicine, Maastricht University Medical Centre (MUMC), Maastricht, The Netherlands; 4 Institute of Molecular Pathobiochemistry, Experimental Gene Therapy and Clinical Chemistry, RWTH-University Hospital Aachen, Aachen, Germany; 5 Department of Hepatology and Gastroenterology, Charité University Medical Center, Berlin, Germany

**Keywords:** MR-proANP, Mid-regional pro atrial natriuretic peptide, ICU, Critical illness, Sepsis, Inflammation, Metabolism, Adipocytokines, Diabetes, Obesity

## Abstract

**Background and aims:**

Mid-regional pro atrial natriuretic peptide (MR-proANP) is an established biomarker for heart failure, based on its key role in regulating homeostasis of water balance and blood pressure. The aim of the study was to determine the value of MR-proANP as a clinical biomarker in critical illness and/or sepsis. Upon admission to the medical intensive care unit (ICU), we investigated MR-proANP plasma concentrations in 217 critically ill patients (144 with sepsis, 73 without sepsis). Results were compared with 65 healthy controls.

**Results:**

MR-proANP plasma levels were significantly elevated in critically ill patients, when compared to healthy controls. Notably, MR-proANP levels were significantly higher in ICU patients with sepsis. MR-proANP levels were not associated with metabolic comorbidities like diabetes or obesity. In critically ill patients, MR-proANP plasma concentrations correlated with inflammatory cytokines, markers of organ dysfunction and several adipocytokines, such as resistin, retinol-binding protein 4 (RBP4) and adiponectin. Importantly, high MR-proANP plasma levels were associated with mortality, as MR-proANP levels above 227.0 pmol/l indicated a particularly increased mortality risk in ICU patients. The association between MR-proANP and mortality was independent of single organ failure and inflammation markers.

**Conclusion:**

Our study emphasizes the role of circulating MR-proANP as a biomarker in critically ill patients, in which high MR-proANP indicates organ dysfunction, sepsis and mortality risk. The association between high MR-proANP and inflammatory as well as adipose tissue-derived endocrine mediators warrants further pathophysiological investigations.

## Background

The natriuretic peptides of type A, B and C (ANP, atrial natriuretic peptide; BNP, brain natriuretic peptide; CNP, C-type natriuretic peptide) belong to a family of cardiac- and vascular-derived hormones. They exert diuretic, natriuretic and hypotensive actions and protect the organism from excessive fluid and high blood pressure. Through a variety of effects on vascular tone, intravascular volume and redistribution, cardiovascular remodelling and energy metabolism, natriuretic peptides play a key role in maintaining cardiovascular homeostasis, water balance and blood pressure [1–4]. In this context, atrial natriuretic peptides (ANP) are predominantly expressed in the right atrium of the heart and secreted during an atrial distension such as in cardiac dysfunction or heart failure [1, 5, 6]. ANP represents more than 95% of natriuretic peptides in the blood circulation [7]. It is synthesized and stored in the atrial cardiomyocyte granules as a prohormone with 126 amino acids (proANP 1–126) [8]. The prohormone consists of several peptides with antihypertensive, natriuretic, diuretic and kaliuretic properties (e.g. proANP1–30—long-acting natriuretic peptide; proANP31–67—vasodilator; proANP79–98—kaliuretic peptide) [9]. ANP prohormone processing differs within the kidney leading to an additional addition of four amino acids to the N-terminus of ANP (e.g. proANP95–126). The mature atrial natriuretic peptide consists of amino acids 99–126 and comprises 98% of the circulating natriuretic peptides [10]. In response to increased tension of the atrial wall, the active hormone ANP is secreted by splitting of its precursor into an amino-terminal (NT-proANP 1–98) and an active hormone (ANP 99–126) [7, 11–14]. As the active ANP has a very short half-life of less than 5 min and the NT-proANP is released in the same molar ratio as ANP with significantly longer half-life (60–120 min), NT-proANP is considered a more reliable biomarker than ANP itself [8, 15]. However, since NT-proANP can be further cleaved into smaller amino acid fragments in vivo, the detection of mid-regional proANP (amino acids 53–90; MR-proANP) is the preferred detection site of this natriuretic peptide [10, 16–18].

ANP disrupts both network of mitogen-activated protein kinase (MAPK) and associated transcription factors, mainly NF-κB [5]. Atrial dilatation leads to the expression and secretion of preproatrial or A-type natriuretic peptide and finally atrial natriuretic peptide (ANP), which has similar biological properties as B-type natriuretic peptide (BNP). However, the formation of the pre-produced BNP is induced by sodium and water retention and vasoconstrictions caused by the activation of RAAS and the sympathetic nervous system, as well as the action of vasopressin. These factors lead to increased ventricular pre- and post-stress and increased wall stress and BNP release. Furthermore, the BNP prohormone is cleaved to BNP and N-terminal proBNP (NT-proBNP). NT-proBNP is biologically inactive and does not bind to the pGC-A receptor. However, the biological effects of ANP and BNP substrates are similar: induction of natriuresis, diuresis, vasodilatation, antifibrosis and anti-RAAS [5, 9].

ANP acts through the natriuretic peptide receptor A (NPR-A) and is removed from the bloodstream by the natriuretic peptide receptor C (NPR-C) [7, 19]. Binding to NPR-A activates the cyclic guanylyl monophosphate (cGMP) as second messenger in the target cells to mediate a variety of systemic effects as previously described [19]. Interestingly, in knock-out mice lacking the NPR 1 gene coding for NPR-A, not only high ANP concentrations, hypertension and cardiac hypertrophy, but also expression of pro-inflammatory markers are observed. In line, recent data suggest that ANP regulates inflammatory processes such as macrophage function, priming of neutrophils and the expression of pro-inflammatory markers [20, 21]. Thus, ANP also participates in innate immune reactions [2, 22]. Moreover, activation of intracellular cGMP induces lipolysis and mobilization of free fatty acids in human adipocytes [23, 24]. This demonstrates the interaction of ANP with white and brown adipose tissue. Specifically, ANP increases the expression and secretion of adiponectin, an adipocytokine with insulin-sensitizing properties, as observed in primary human adipocyte cultures, healthy subjects and patients with congestive heart failure [23, 25, 26]. In addition, the ANP/cGMP signalling pathway increases β-cell mass and insulin secretion in the pancreas [23]. With regard to ANP removal, it has been clearly shown that upregulation of NPR-C is associated with metabolic alterations such as obesity and obesity-related metabolic disorders like type 2 diabetes and metabolic syndrome [23, 27].

Based on this wide range of physiological functions of ANP and its associated alterations, ANP has been linked to inflammatory responses and metabolic alterations that occur during critical illness [9, 28, 29]. Critical illness and MR-proANP are associated with and affected by alterations in secretory and metabolic functions of adipose tissue [30, 31]. In different cohorts of ICU patients, high MR-proANP plasma levels have been associated with disease severity and outcome of critical illness [8, 11]. In addition, elevated MR-proANP levels are described to be diagnostic for sepsis after burn injury [32]. In this study, we investigated the clinical and prognostic relevance of MR-proANP plasma concentrations in a large cohort of critically ill patients from a medical ICU including sepsis, pre-existing diabetes, obesity and organ dysfunction.

## Methods

### Study design and patient characteristics

Critically ill patients were included at admission to the medical ICU at the RWTH University Hospital Aachen, Germany. Patients, who were admitted for post-interventional observational stay or underwent an elective procedure, were excluded [33]. The cohort consisted of 217 critically ill patients (144 with sepsis, 73 without sepsis). Patients’ characteristics are shown in Table 1. The patients were categorized as sepsis and non-sepsis according to the Third International Consensus Definitions for Sepsis and Septic Shock (sepsis-3) [34], and were treated following the current guidelines for treatment of sepsis (Surviving Sepsis Campaign) [35]. Underlying disease etiologies of sepsis and non-sepsis patients are shown in Table 2. As a control group, we analysed healthy blood donors with normal blood counts, normal values of liver enzymes, glomerular filtration rates, serum creatinine and C-reactive protein (CRP) concentration [36]. All healthy subjects had a negative serology for human immunodeficiency virus (HIV) [36]. In order to determine long-term outcome, we contacted the patients, their relatives and/or the general practitioner in approximately 6-months intervals after discharge from the hospital over a period of 3 years [36].

**Table 1:** Baseline patient characteristics and MR-proANP plasma concentrations

Parameter	All patients	Non-sepsis	Sepsis
Number	217	73	144
Sex (male/female)	133/84	48/25	85/59
Age median (range) [years]	64 (18–90)	61 (18–85)	65 (20–90)
APACHE-II score median (range)	18 (2–43)	13.5 (2–33)	19 (4–43)
SOFA score median (range)	9 (0–19)	7.0 (0–17)	9.5 (2–19)
SAPS2 score median (range)	41 (0–73)	41.0 (13–72)	40.5 (0–73)
ICU days median (range)	7 (1–137)	6 (1–45)	9 (1–137)
Death during ICU n(%)	46 (21.2%)	9 (12.3%)	37 (25.7%)
Death overall (total) n(%)	86 (39.6%)	22 (30.1%)	64 (44.4%)
Mechanical ventilation n(%)	144 (66.4%)	46 (63%)	98 (67%)
Preexisting diabetes n(%)	65 (30.0%)	22 (30.1%)	43 (29.9%)
BMI median (range) [m^2^/kg]	26.0 (15.3–86.5)	25.7 (15.9–40.5)	26.0 (15.3–86.5)
WBC median (range) [×10^3^/µl]	12.9 (0.1–208)	12.5 (1.8–29.6)	13.8 (0.1–208)
CRP median (range) [mg/dl]	103.0 (5–230)	17 (5–230)	163.5 (5–230)
IL-6 median (range) [pg/ml]	145.0 (2–28,000)	66.5 (1.5–5000)	240 (2–28,000)
Procalcitonin median (range) [pmol/l]	0.7 (0.03–207.5)	0.2 (0.03–100)	1.8 (0.03–207.5)
Creatinine median (range) [mg/dl]	1.3 (0.1–15)	1.0 (0.2–15)	1.6 (0.1–10.7)
INR median (range)	1.16 (0.92–13)	1.17 (0.95–6.73)	1.16 (0.92–13)
MR-proANP day 1 median (range) [pmol/l]	214.0 (2.1–3417.0)	147.2 (2.1–1625.0)	246.6 (7.8–3417.0)

For quantitative variables, median and range (in parenthesis) are given

**Table 2:** Disease etiology of the study population leading to ICU admission

	Sepsis, n (%), n = 144	Non-sepsis, n (%), n = 73
Etiology of sepsis critical illness		
Site of infection		
Pulmonary	73 (51%)	
Abdominal	26 (18%)	
Urogenital	11 (8%)	
Other	34 (23%)	
Etiology of non-sepsis critical illness		
Cardio-pulmonary disorder		29 (40%)
Acute pancreatitis		10 (14%)
Acute liver failure		4 (5.5%)
Decompensated liver cirrhosis		9 (12%)
Severe gastrointestinal hemorrhage		4 (5.5%)
Non-sepsis other		17 (23%)

### Measurements of MR-proANP plasma levels

Before therapeutic interventions, blood samples were collected upon admission to the ICU, centrifuged, and plasma was stored at − 80 °C. Plasma MR-proANP concentrations (epitopes covering amino acids 53–90, equivalent to NT-proANP and active ANP [10] were determined using an automated immunofluorescent assay based on TRACE technology (Time-resolved Amplified Cryptate Emission, B.R.A.H.M.S Kryptor compact, Hennigsdorf, Germany), according to manufacturer`s instructions (MR-proANP Kryptor, #819.050, B.R.A.H.M.S, Hennigsdorf, Germany). Measurements of the adipocytokines and related proteins leptin, retinol-binding protein 4 (RBP4), adiponectin, ghrelin, and resistin were included, as previously reported [37–41]. In addition, soluble urokinase-type plasminogen activator receptor (suPAR) and amino-terminal pro C-type natriuretic peptide (NT-proCNP) concentrations as markers of disease severity and inflammatory response were also investigated as described previously [36, 42].

### Statistical analysis

Owing to the skewed distribution of the parameters, data are given as median and range, and shown graphically by box-and-whiskers plots. The degree of association between two variables was assessed by the Spearman rank correlation test. Comparisons of parameters between two different groups were conducted with the Mann–Whitney U-test. All values, including outside values as well as far out values, were included. P-values less than 0.05 were considered as statistically significant. Receiver operating characteristic (ROC) curve analysis was carried out to determine the diagnostic sensitivity and specificity of MR-proANP in critically ill patients. The ROC curve analysis and the derived area under the curve (AUC) statistic provide a global and standardized appreciation of the accuracy of a marker or a composite score for predicting an event. ROC curves were generated by plotting sensitivity against 1-specificity [42]. The prognostic value of the variables was tested by univariate and multivariate analyses in the Cox regression model. Survival curves were generated by Kaplan–Meier analyses with an MR-proANP cut-off level calculated via the Youden Index [42]. All analyses were performed with IBM SPSS Statistics (SPSS; Chicago, IL, USA).

## Results

### MR-proANP plasma levels are significantly elevated in critically ill patients as compared with healthy controls

**Fig. 1:**
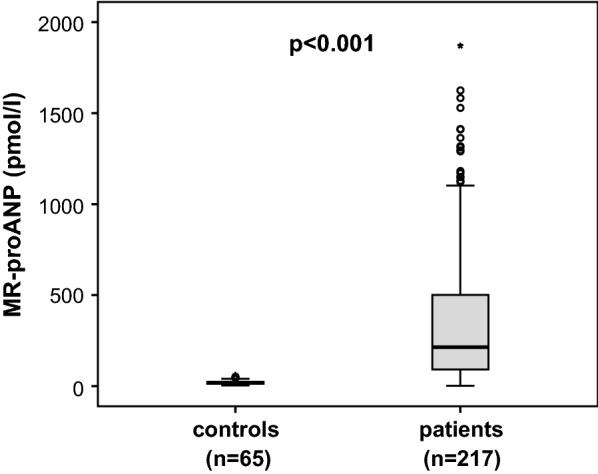
MR-proANP levels in critically ill patients MR-proANP plasma concentrations are significantly elevated in critically ill patients compared with healthy controls. *p* value (U-test) is given

Based on the wide range of physiological functions of ANP and its associated alterations, ANP has been linked to both inflammatory and metabolic responses that typically occur during critical illness [9, 28, 29]. In our study, we found that MR-proANP plasma levels were significantly elevated in a large cohort of 217 critically ill patients (median 214.0 pmol/l, range 2.1–3417.0 pmol/l; Table 1) at admission to the ICU as compared with 65 healthy controls (median 18.5 pmol/l, range 3.5–61.7 pmol/l, p < 0.001; Fig. 1).

### MR-proANP plasma levels are particularly elevated in critically ill patients with sepsis

**Fig. 2:**
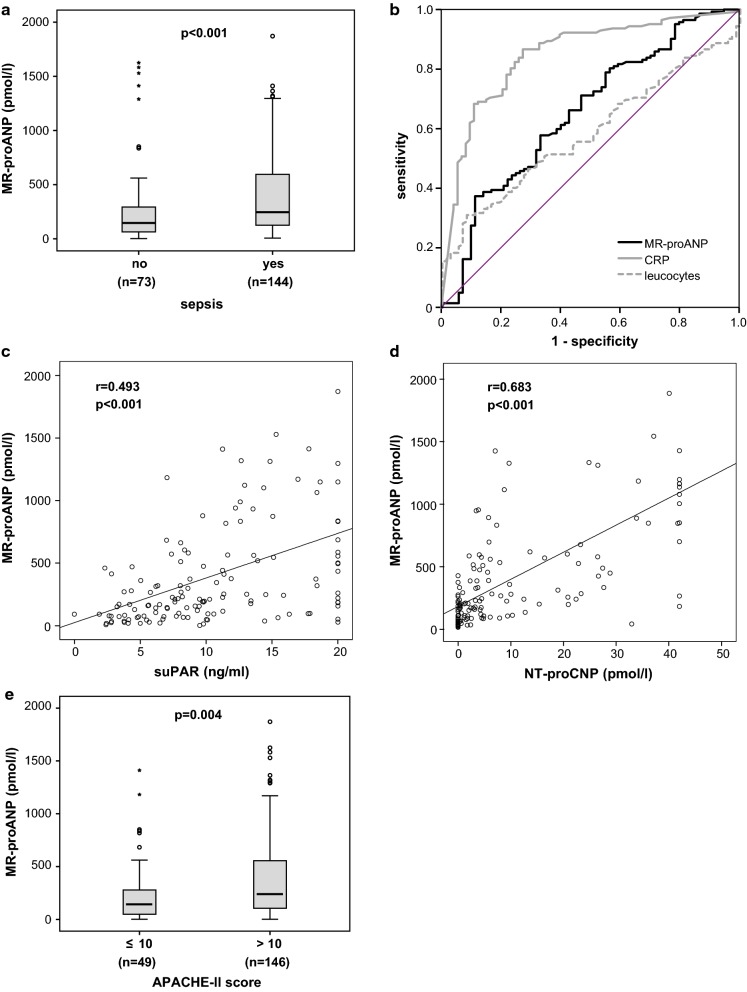
MR-proANP levels are elevated in critically ill patients with sepsis and correlate with inflammatory markers **a** ICU patients with sepsis displayed significantly elevated MR-proANP levels compared to patients without sepsis. **b** Receiver operating characteristic (ROC) curve analyses comparing the diagnostic power in predicting sepsis of MR-proANP (black line, area under the curve (AUC) 0.656) with classical markers of inflammation and bacterial infection, C-reactive protein (CRP, grey line, AUC 0.847) and white blood cell count (leukocytes, dotted grey line, AUC 0.585). **c–d** MR-proANP correlates with experimental markers of inflammation in critical illness like soluble urokinase-type plasminogen activator receptor (suPAR, C) and N-terminal pro C-type natriuretic peptide (NT-proCNP, D). **e** At ICU admission, MR-proANP levels are significantly elevated in critically ill patients with high initial Acute Physiology and Chronic Health Evaluation (APACHE II) score (> 10) in comparison to patients with low APACHE-II scores (≤ 10). p-values (U-test or Spearman rank correlation) are given

High MR-proANP plasma levels in critically ill patients had been previously reported to be associated with sepsis [8, 11]. Within our cohort of 217 critically ill patients, 144 fulfilled sepsis criteria, while 73 were admitted to the ICU due to other causes of critical illness (Table 2). Plasma concentrations of MR-proANP were significantly elevated in patients with sepsis (median 246.6 pmol/l, range 7.8–3417.0 pmol/l) as compared to ICU patients without sepsis (median 147.2 pmol/l, range 2.1–1625.0 pmol/l, p < 0.001; Fig. 2a and Table 2). We analysed the diagnostic value of MR-proANP for sepsis in comparison to classical markers of inflammation and bacterial infection by using ROC curve analyses. Whereas CRP achieved AUC statistics of 0.847 and white blood cell count of 0.585, MR-proANP only reached an AUC of 0.656 (Fig. 2b). Among the septic or non-septic critically ill patients, there was no association between MR-proANP plasma concentrations and different disease etiologies leading to ICU admission (data not shown).

### MR-proANP levels in critically ill patients are closely correlated to biomarkers of inflammation, organ dysfunction and clinical scores

Mice lacking a functional NPR 1 gene encoding NPR-A exhibit hypertension and marked cardiac hypertrophy with interstitial fibrosis, in association with enhanced activation of pro-inflammatory cytokines, probably via nuclear factor kappa mediated signalling pathway [43, 44]. To determine the factors possibly promoting elevated MR-proANP plasma levels in critically ill patients, correlation analyses with extensive sets of laboratory parameters were performed. At admission to the ICU, plasma MR-proANP concentrations in the total cohort and the subgroups of sepsis and non-sepsis patients were closely correlated with classical markers of inflammation and bacterial infection, such as C-reactive protein (r = 0.286, p < 0.001), procalcitonin (r = 0.378, p < 0.001), and experimental markers of inflammation such as soluble urokinase plasminogen activator receptor (suPAR, r = 0.493, p < 0.001, Fig. 2c), and NT-proCNP (r = 0.683, p < 0.001, Fig. 2d, Table 3).

**Table 3:** Correlations with MR-proANP plasma concentrations at ICU admission

Parameters	ICU patients	
	R	*p*
Disease severity/clinical scoring/therapy		
APACHE II	0.260	< 0.001
SOFA	0.223	0.011
SAPS	0.341	0.006
Fluid substitution	− 0.233	0.001
Markers of inflammation		
White blood cell count	− 0.148	0.029
C-reactive protein	0.286	< 0.001
Procalcitonin	0.378	< 0.001
suPAR	0.493	< 0.001
NT-proCNP	0.683	< 0.001
Markers of organ function		
NT-proBNP	0.740	< 0.001
Urea	0.623	< 0.001
Creatinine	0.629	< 0.001
GFR-cystatin C	− 0.675	< 0.001
Cystatin C	0.675	< 0.001
Lipase	− 0.191	0.012
Pancreatic amylase	− 0.317	0.006
Alanine aminotransferase	− 0.172	0.012
Glutamate dehydrogenase	− 0.151	0.037
Pseudocholinesterase activity	− 0.339	< 0.001
Albumin	−0.190	0.045
Total protein	− 0.263	< 0.001
INR	0.207	0.003
aPTT	0.324	< 0.001
Antithrombin III	− 0.216	0.015
Adipocytokines/metabolic markers		
Adiponectin	0.434	0.001
Resistin	0.349	0.008
RBP4	0.306	0.012
HOMA-β	0.332	0.007
Parathyroid hormone	0.299	0.014
Calcium	− 0.288	< 0.001
Phosphorus	0.241	0.001

Spearman rank correlation test, only significant results are shown

With regard to organ function, we could reveal strong associations with renal and hepatic function for the total study cohort and the subgroups of sepsis and non-sepsis patients. Specifically, we could demonstrate an inverse association with renal function as displayed by a highly significant correlations with the glomerular filtration rate of cystatin C (r = − 0.675, p < 0.001), cystatin C (r = 0.675, p < 0.001), creatinine (r = 0.629, p < 0.001) and urea (r = 0.623, p < 0.001) serum concentrations (Table 3), indicating renal clearance of MR-proANP [45]. Interestingly, MR-proANP levels inversely correlated with parameters reflecting the liver’s biosynthetic and functional capacity, namely albumin (r = − 0.190, p = 0.045), pseudocholinesterase activity (r = − 0.339, p < 0.001), antithrombin III (r = − 0.216, p = 0.015), glutamate dehydrogenase (r = − 0.151, p = 0.037) and alanine aminotransferase (r = − 0.172, p = 0.012) (Table 3). MR-proANP levels also correlated with the amino-terminal brain natriuretic peptide (NT-proBNP) (Table 3).

Increased MR-proANP levels have been associated with adverse clinical outcome [46]. In fact, MR-proANP plasma levels correlated positively with established clinical disease severity scores (Table 3). Moreover, critically ill patients with a high (acute physiology and chronic health II (APACHE-II) score above 10 showed significantly higher MR-proANP levels at ICU admission (median 239.7 pmol/l, range 2.1–1871.0 pmol/l) in comparison to ICU patients admitted with an APACHE-II score of 10 or less (median 143.0 pmol/l, range 2.1–3417.0 pmol/l, p = 0.004, Fig. 2e).

For the total cohort of critically ill patients a strong association of MR-proANP plasma concentrations and established clinical scores like sequential organ failure assessment (SOFA; r = 0.223, p = 0.011), simplified acute physiology score 2 (SAPS2; r = 0.341, p = 0.006), and acute physiology and chronic health II (APACHE II; r = 0.260, p < 0.001) scores could be shown, corroborating that MR-proANP levels are closely linked to disease severity in critical illness (Table 3).

Measures of hemodynamic instability such as need for volume substitution and vasopressor therapy showed a significant inverse correlation of fluid therapy with plasma MR-proANP levels (r = − 0.233, p = 0.001), but not with vasopressor administration (Table 3).

### MR-proANP plasma levels in critically ill patients are not associated with diabetes and obesity

**Fig. 3:**
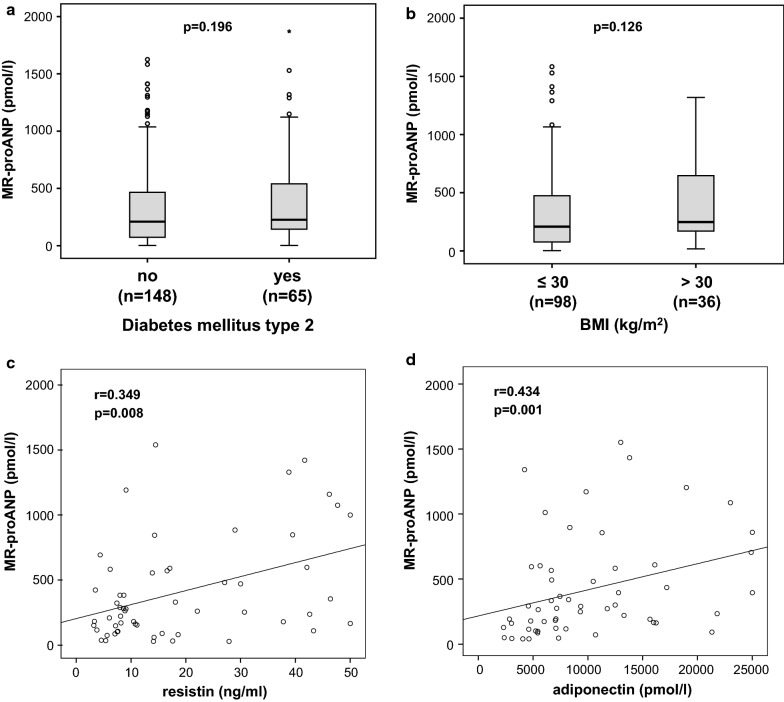
MR-proANP levels are not related to metabolic comorbidities **a–b** MR-proANP plasma concentrations in ICU patients are neither associated with pre-existing type 2 diabetes **a** nor obesity, as defined by a body-mass index (BMI) above 30 kg/m^2^**b**. **c–d** We observed significant correlations between MR-proANP and several adipocytokines including resistin (**c**) and adiponectin (**d**). p-values (U-test or Spearman rank correlation) are given

Prior studies have shown an inverse association with natriuretic peptides and metabolic syndrome, fasting glucose, insulin resistance and diabetes development [45, 47]. We therefore assessed whether metabolic comorbidities, specifically pre-existing obesity or diabetes, might have an influence on MR-proANP levels also in patients with critical illness. However, neither pre-existing type 2 diabetes (n = 65, median 226.6 pmol/l, range 2.1–1871.0 pmol/l, p = 0.196; Fig. 3a) nor obesity (n = 36, median 248.1 pmol/l, range 17.5–1319.0 pmol/l, p = 0.126; Fig. 3b), as defined by a body mass index (BMI) above 30 kg/m^2^, were associated with MR-proANP plasma concentrations. Moreover, by Spearman rank correlation analysis, no correlation between MR-proANP and serum glucose levels, glycosylated hemoglobin A1c (HbA1c) or BMI was present (data not shown). In addition, MR-proANP did not show any correlations with other key markers of glucose metabolism, such as insulin, C-peptide or the homeostasis model assessment-insulin resistance (HOMA-IR) in ICU patients (data not shown). However, β-cell function (HOMA-β) correlated with MR-proANP (r = 3.332, p = 0.007, Table 3).

Adipose tissue inflammation attributes to dysregulated production and release of inflammatory cytokines and adipocytokines, including interleukin-6 (IL-6), tumor necrosis factor-α (TNF-α) as well as leptin, resistin and adiponectin [48]. We investigated the potential association between MR-proANP and adipocytokine responses in critically ill patients. In agreement with the proposed pro-inflammatory association of MR-proANP, we observed significant correlations between MR-proANP and a broad range of adipocytokines including resistin (r = 0.349, p = 0.008, Fig. 3c), adiponectin (r = 0.434, p = 0.001, Fig. 3d), and RBP4 (r = 0.306, p = 0.012, Table 3).

### Elevated MR-proANP plasma levels are associated with mortality in critically ill patients

**Fig. 4:**
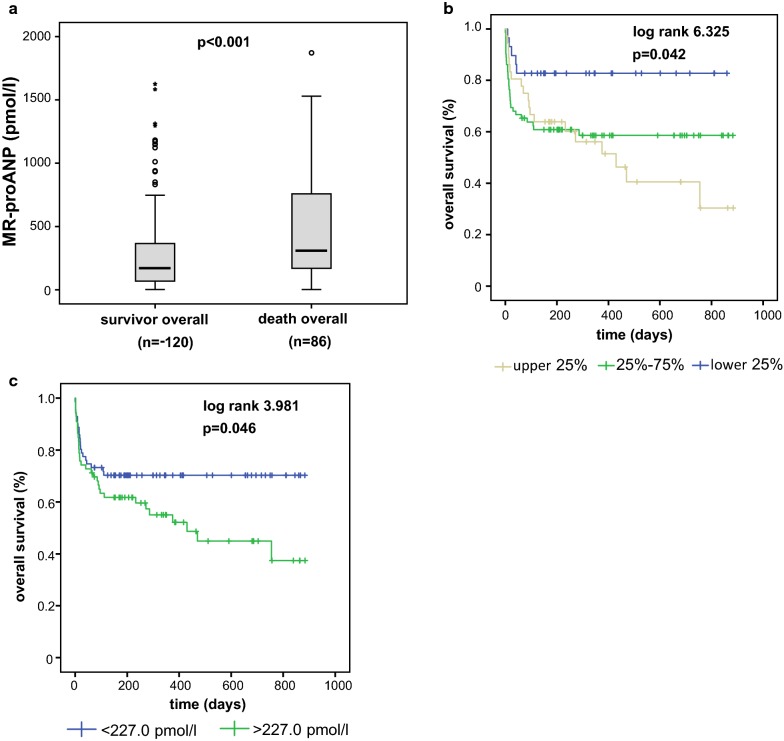
MR-proANP is a biomarker for mortality in critically ill patients **a** Patients that died during or after ICU treatment displayed significant higher MR-proANP levels at ICU admission compared to patients that survived in the long-term follow-up. **b** High vs. low MR-proANP plasma concentrations discriminated survival of the critically ill patients, as displayed by Kaplan–Meier survival curve analysis for MR-proANP separated into quartiles. **c** Elevated MR-proANP plasma concentrations at ICU admission (optimal cut-off: 227.0 pmol/l) predicted the overall mortality in critically ill patients. p-values (U-test or log rank test) are given

Circulating natriuretic peptides like NT-proCNP have been previously suggested as biomarkers for disease severity as well as short- and long-term survival in various conditions of critical illness [42]. We assessed long-term survival in 206 out of 217 patients by contacting the patients, their relatives or their general practitioner during the first three years after ICU discharge. MR-proANP levels at ICU admission were significantly elevated in patients that subsequently died (n = 86, median 309.0, range 2.1–3417.0) compared with survivors (n = 120, median 171.1, range 2.1–1625.0; p < 0.001, Fig. 4a).

By univariate analysis, including markers of inflammation/infection (CRP, p = 0.111; lactate, p = 0.198), hepatic (bilirubin, p = 0.161) and renal (creatinine, p = 0.427) function at admission were not significantly associated with mortality, while MR-proANP showed highest prognostic value (p = 0.013) for ICU mortality. In multivariate Cox regression analyses (including the above mentioned parameters in the model) MR-proANP remained an independent and the only significant prognostic parameter (p = 0.012) to predict overall ICU mortality. In this respect, MR-proANP levels showed comparable prognostic accuracy like established multifactorial scores such as APACHE II (AUC = 0.654 for MR-proANP, 0.638 for APACHE II score changes in ROC analyses). This finding was corroborated by Kaplan–Meier survival curves analyses, demonstrating that patients with MR-proANP plasma levels of the lower quartile (< 25%, corresponding to 91.3 pmol/l) had the best survival rates, while patients with admission MR-proANP levels of the upper quartile (> 75%, corresponding to 506.6 pmol/l) had the highest long-term mortality (Fig. 4b). Using the calculated optimal cut-off for MR-proANP of 227.0 pmol/l, patients with high MR-proANP demonstrated a high mortality rate, as depicted by Kaplan–Meier survival curve analysis (Fig. 4c).

## Discussion

The expression and secretion of the atrial natriuretic polypeptide (ANP) hormone has been mainly studied in the context of cardiac diseases [49]. In particular, increases in ANP or MR-proANP concentrations in blood circulation were often considered to be dependent on the prevalence of cardiac insufficiency and classical cardiac risk factors such as diabetes and renal failure [50]. Furthermore, ANP has been linked to both inflammatory and metabolic responses that typically occur during critical illness [9, 28, 29].

However, ANP is expressed and secreted by the cells of the heart atria and BNP, mainly in the ventricles that is therefore less sensitive to intraventricular pressure increase and hemodynamic stress than BNP. NT-proBNP is currently recognized as the clinical gold standard for the diagnosis of acute destabilized heart failure in patients with dyspnea [51]. In critically ill patients, elevated plasma concentrations of natriuretic peptides are found in severe hemodynamic disturbances such as cardiogenic or septic shock due to ventricular dysfunction and the release of proinflammatory cytokines [5, 9]. In accordance to the positive correlation between MR-proANP and NT-pro BNP in our study, dramatically increased proinflammatory cytokines in critically ill patients may also contribute to ANP and BNP secretion from the heart.

In our study, we demonstrated that MR-proANP is elevated in critically ill patients already at admission to the ICU as compared with healthy controls, in agreement with prior studies [8, 11, 32]. Moreover, using correlation analyses our study revealed significant associations between MR-proANP and established biomarkers reflecting inflammation, metabolic alterations, and organ dysfunction in medical ICU patients.

Although MR-proANP levels were further elevated in critically ill patients with sepsis, their diagnostic power for sepsis was inferior to routinely used inflammatory markers such as CRP or procalcitonin. In line with our findings, it has been reported that MR-proANP is neither a direct sepsis marker nor a predictor of bacteraemia [32, 52, 53]. Interestingly, in ventilator-associated pneumonia and lower respiratory tract infections, implementing MR-proANP improved survival prediction of clinical severity scores, especially when used in combination with procalcitonin (PCT) [54, 55]. In septic shock patients, MR-proANP was significantly associated with 28-day mortality [56]. Moreover, MR-proANP was associated with cardiorenal dysfunction and an increased risk of terminal kidney disease and mortality [57]. In this context, MR-proANP showed a high accuracy for predicting survival in critical ill patients in our study.

Several studies have shown that obese individuals display lower circulating natriuretic peptide concentrations, indicating that obesity or BMI may be confounding factors for clinical and prognostic utility of MR-proANP [58–60]. In our cohort, we found that MR-proANP is strongly correlated with adipocytokines such as adiponectin, RBP4 and resistin, which are important mediators of insulin resistance and metabolic alterations [36, 42]. Interestingly, MR-proANP did only correlate with markers reflecting adipose tissue inflammation, but not with patient’s BMI or pre-existing obesity. Critically ill patients show dramatic metabolic and inflammatory dysfunctions, including dysregulated adipocytokines [30, 31]. Within this context, ANP-binding to the natriuretic peptide receptor A activates the cyclic guanylyl monophosphate (cGMP) to mediate a variety of systemic effects such as lipolysis and free fatty acid mobilization in human adipocytes [19, 23, 24], which may provoke adipocytokine secretion from adipose tissue. The effects of ANP on adipose tissue might sustain inflammatory responses, possibly supporting systemic inflammation in critical illness and sepsis. Our findings demonstrate the potential diagnostic and prognostic value of MR-proANP in critically ill patients with sepsis and may contribute to implement MR-proANP as a potential novel biomarker in critical disease.

## Conclusion

Our study emphasizes the role of circulating MR-proANP as a potential novel biomarker in critically ill patients, in which high MR-proANP plasma concentrations indicate organ dysfunction, sepsis, disease severity and mortality risk. The association between high MR-proANP and inflammatory as well as adipose tissue-derived endocrine mediators warrants further pathophysiological investigations. Knowledge of these interactions will enhance the understanding of the pathogenic role of natriuretic peptides in critical illness.

## Data Availability

The datasets used and/or analysed during the current study are available from the corresponding author on reasonable request.

## Abbreviations

ANP: atrial natriuretic peptide
APACHE: acute physiology and chronic health evaluation
BMI: body mass index
BNP: brain natriuretic peptide
cGMP: cyclic guanylyl monophosphate
CNP: C-type natriuretic peptide
GFR: glomerular filtration rate
HbA1c: glycosylated hemoglobin A1c
HIV: human immunodeficiency virus
HOMA-β: homeostasis model assessment-β-cell function
HOMA-IR: homeostasis model assessment-insulin resistance
ICU: intensive care unit
IL-6: interleukin 6
INR: international normalized ration
MR: mid-regional
NPR: natriuretic peptide receptor
NT: amino-terminal
PCT: procalcitonin
RBP4: retinol-binding protein 4
SAPS: simplified acute physiology score
SOFA: sequential organ failure assessment
suPAR: soluble urokinase-type plasminogen activator receptor
TRACE: time-resolved amplified cryptate emission
TNF-α: tumor necrosis factor-α
WBC: white blood cell count
